# Postoperative Imaging of Complications Following Cranial Implants

**DOI:** 10.5334/jbsr.1881

**Published:** 2019-12-20

**Authors:** Chi Long Ho, Louis McAdory

**Affiliations:** 1 Sengkang General Hospital, SG; 2 Duke-National University of Singapore (NUS) Medical School, SG; 3 Department of Diagnostic Radiology, Singapore General Hospital, SG

**Keywords:** Artifacts, cranioplasty, endovascular embolization materials, cranial implants, titanium, ventriculoperitoneal shunts

## Abstract

A wide range of neurosurgical implants, cranioplasty materials and catheters have been developed to treat a variety of intracranial disorders. Interpretation of postoperative imaging can be challenging and confounded by postoperative changes and implant-related complications. Review of recent literature suggested that there is a paucity of data on postoperative cranial implant-related complications.

If not addressed appropriately in a timely manner, these complications may cause a delay in the patient’s treatment with subsequent prolongation of hospital stay. It is therefore paramount for clinicians and radiologists to be aware of the appearance of these implant-related complications on imaging during postoperative surveillance.

## Introduction

Various neurosurgical implants and catheters have been developed to treat a variety of intracranial pathologies. Interpretation of postoperative imaging can be challenging due to postoperative changes and implant-related complications. Postoperative complications can be divided according to the period of occurrence: Immediate (<6 hours), early (between 6–72 hours) or late (>72 hours) following surgery [1]. In this article, we highlight the imaging features of commonly used implants in neurosurgery along with postoperative changes and complications.

### Burr hole cover (Figure 1)

**Figure 1:**
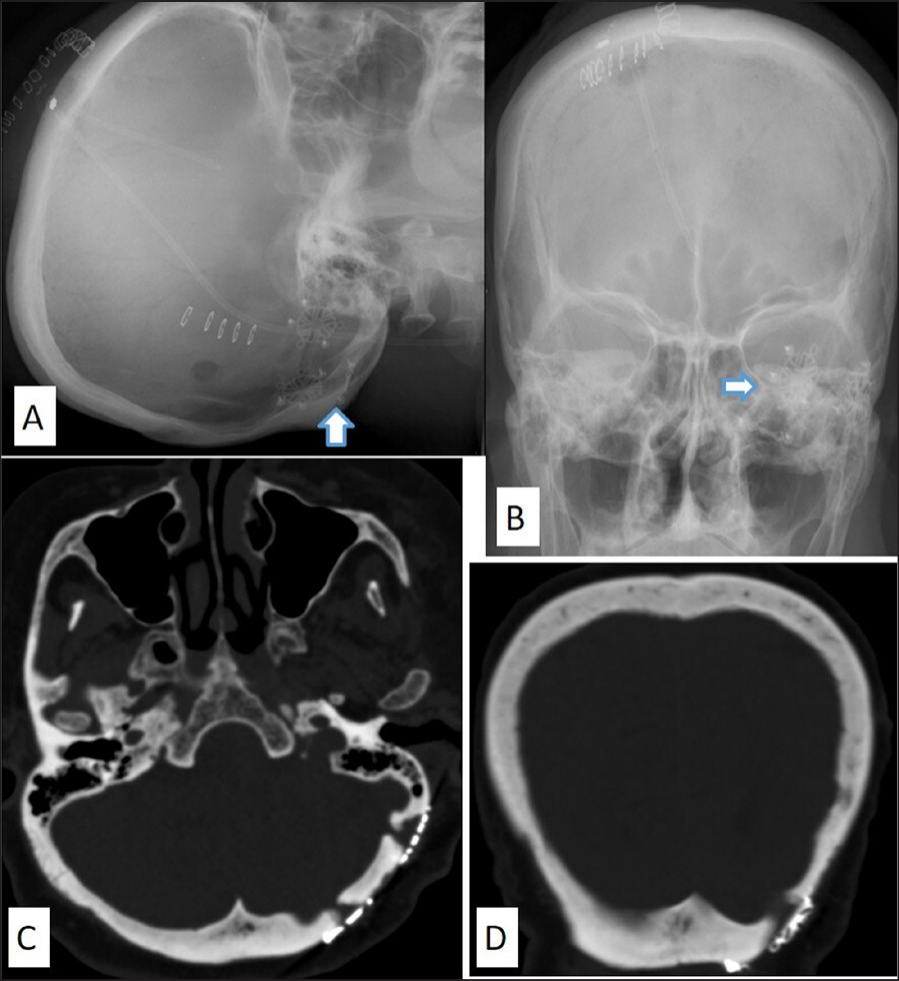
Lateral and anteroposterior skull radiographs **(A)** and **(B)** a 55-year-old man who underwent craniotomy shows two metallic cover plates or craniofix (Aesculap) on the left side of the posterior fossa (A, arrows). Axial CT on skull window **(C)** and **(D)** of the same man shows the metallic cover plates.

A burr hole is created in the skull for evacuation of subdural hemorrhage or stereotactic tumor biopsy. After surgery, the burr hole can be repaired by applying patient’s autologous bone dust or titanium cover plates to prevent sinking of the overlying skin.

### Cranioplasty

Craniotomy is an operation for temporary removal of a bone flap to expose the underlying brain. Titanium CranioFix (Aesculap) plates are widely used to fix the bone flap to the cranium after craniotomy. Rarely, late complication may occur due to skin erosion overlying these plates. Patients with poorly vascularized or fragile skin are particularly vulnerable, risking infections such as meningitis and subdural empyema.

**Figure 2:**
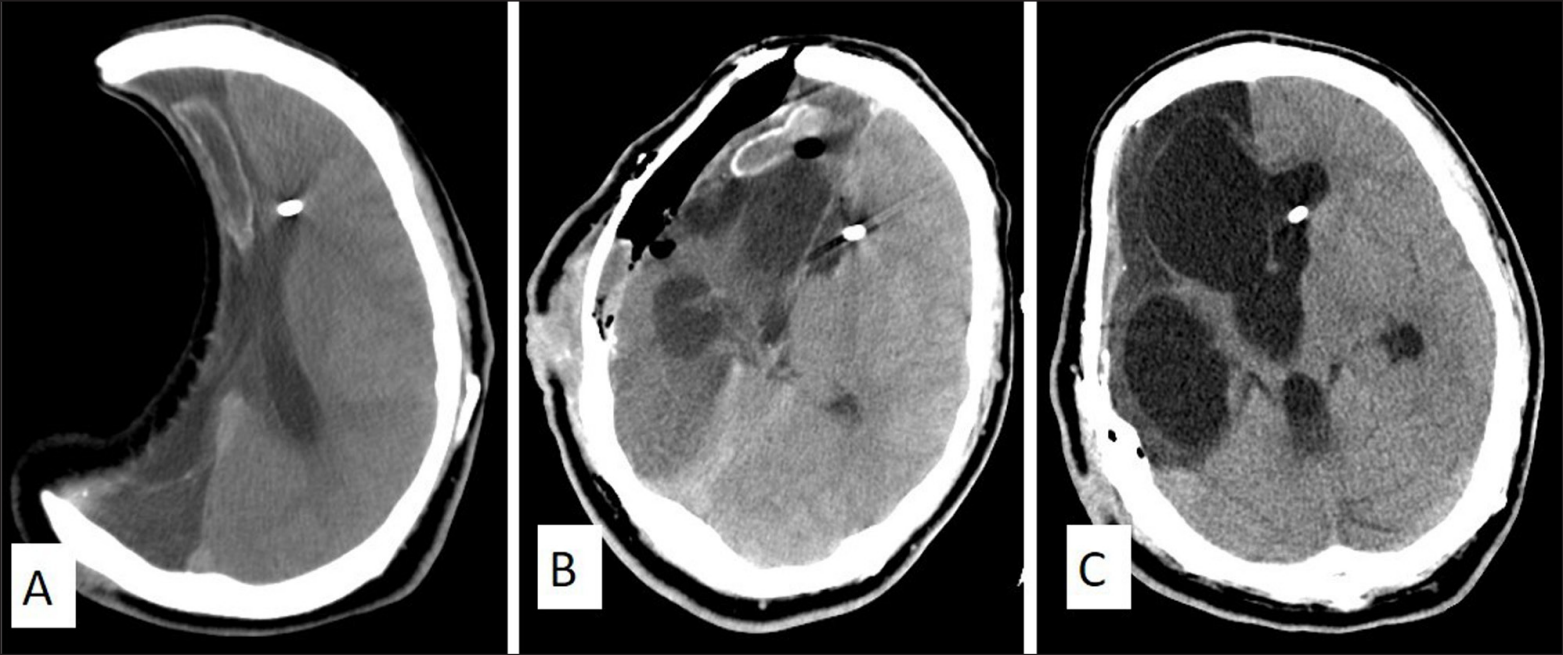
Axial brain CT of a 22-year-old man who underwent right hemicraniectomy. After resolution of the cerebral edema and mass effect, the pathological side of the brain appears “sunken” **(A)**. This appearance is also known as “Motor Trephine Syndrome (MTS)” or “Sunken Brain and Scalp Flap Syndrome” (SSFS). This condition is further aggravated by CSF diversion procedures such as ventriculo-peritoneal shunt. Note of the ventricular catheter tip in the left frontal horn. He subsequently underwent titanium cranioplasty with pneumocephalus underneath the titanium implant **(B)**. There is interval improvement following cranioplasty with improvement of the patients’ neurological and cognitive functions. A few weeks later, he developed a late postoperative complication with hydrocephalus due to shunt dysfunction **(C)**.

Craniectomy is a similar surgical approach as craniotomy except that the skull flap is not replaced after surgery (Figure 2A). It is commonly used to treat medically intractable raised intracranial pressure (ICP) secondary to either malignant infarction or intracranial hemorrhage. A late complication following craniectomy is the “sinking” of the skin flap over the surgical site, known as the “Sunken brain and Scalp Flap Syndrome”(SSFS) or “Motor Trephine Syndrome” (MTS) (Figure 2A). These patients present with sensorimotor deficit and neurological deterioration, which can be aggravated by CSF diversion procedures such as ventriculoperitoneal (VP) shunts (Figure 2A and 2B). According to some authors [2], patients with MTS or SSFS may benefit from an early cranioplasty, which has been shown to improve the “sunken” brain with subsequent improvement of patients’ neurological and cognitive functions (Figure 2B). Early cranioplasty for MTS may therefore serve as a therapeutic procedure rather than being merely cosmetic [2].

**Figure 3:**
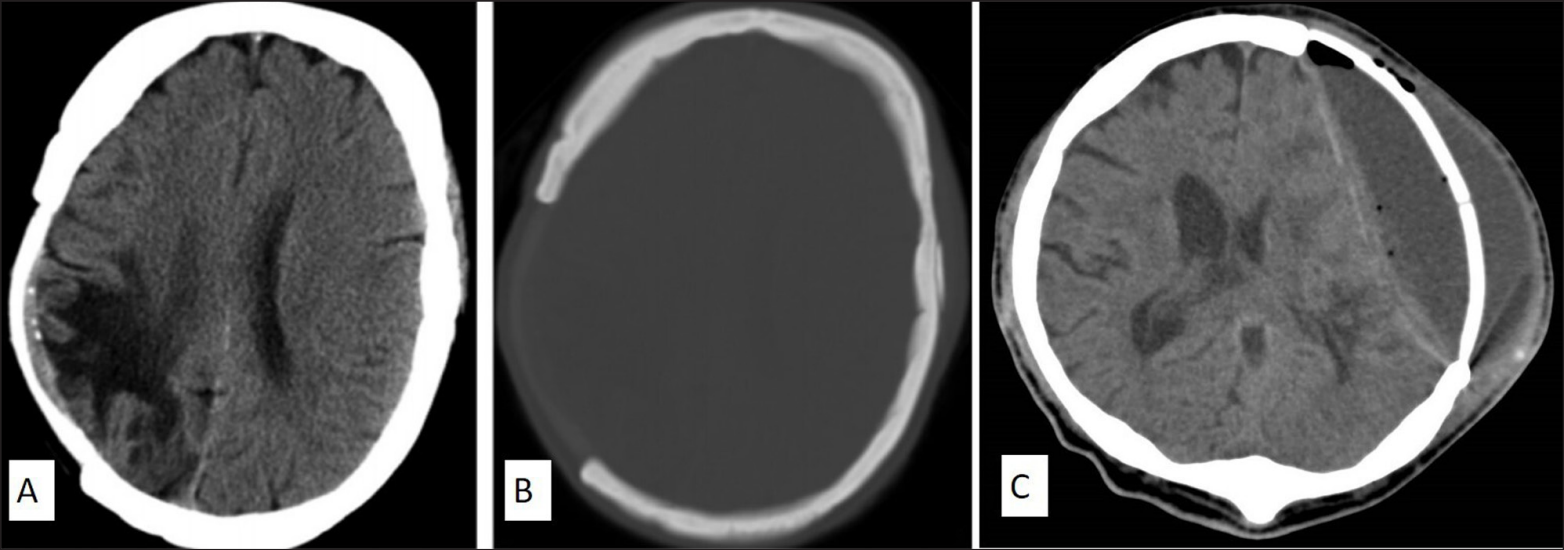
Axial CT, brain window **(A)** and skull bone window **(B)** images of a 50-year-old man demonstrate a right temporo-parietal acrylic cranioplasty using methylmethacrylate (MMA) material which is prefabricated and created with computer-aided design 3D CT data set of the skull defect. Acrylic cranioplasty appears radiolucent on CT and occasionally with mixed intermediate and low attenuation. It may contain gas bubbles formed during exothermic polymerization hence, it should not be mistaken for infection. **(C)** Is from a 48-year-old man with increasing scalp swelling following insertion of a polyetheretherketone (PEEK) cranioplasty with accumulation of large amount of extra-axial fluid collection containing gas on either side of the PEEK cranioplasty and causing mass effect on the underlying cerebrum. There was suspicion of infection of the affected PEEK cranioplasty, which was subsequently removed. The culture grew staphylococcus aureus. The patient recovered gradually following treatment with appropriate intravenous antibiotics.

**Figure 4:**
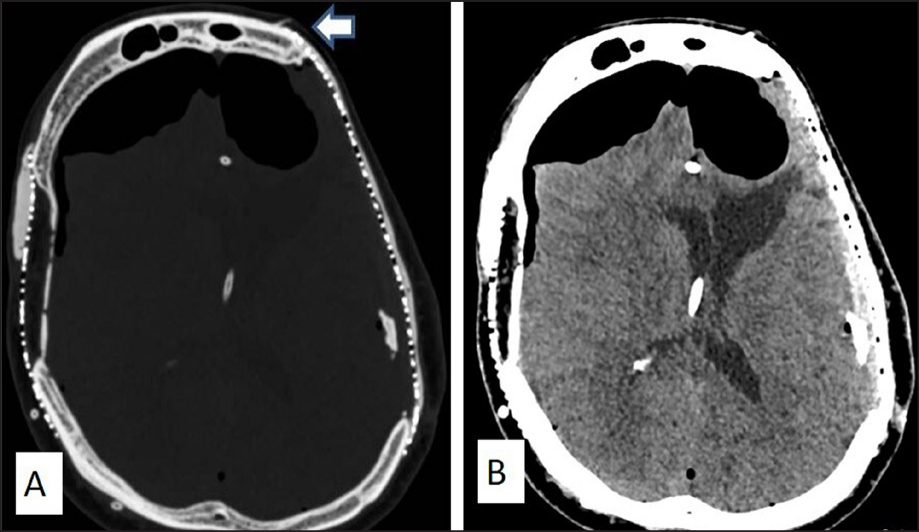
Axial brain CT of a 68-year-old man with bilateral titanium cranioplasty, who developed pneumocephalus five years after surgery due to erosion of the titanium plate through a left frontal scalp wound **(A**, arrow**)**. Pneumocephalus compressed both frontal lobes resulting in a tented appearance of the brain known as the Mount Fuji sign, indicative of tension pneumocephalus **(B)**. After removal of the titanium plate, the scalp was reconstructed with a rotation flap and skin graft.

The material used for cranioplasty are either patient’s autologous bone flap, titanium mesh (Figures 2 and 4), customized acrylic (Figure 3A and 3B) or poly-ether-ether-ketone (PEEK) (Figure 3C).

The most commonly used acrylic resin is methylmethacrylate (MMA), which is prefabricated and created with computer-aided 3D CT data of the skull defect. Acrylic cranioplasty may appear radiolucent on CT and contain gas bubbles formed during exothermic polymerization hence, it should not be mistaken for infection (Figure 3A and 3B). Titanium mesh is one of the most widely used materials for calvarial reconstruction as it is considered safe and avoids cosmetic deformity.

Immediate postoperative complications following cranioplasties are intracranial hemorrhages and infections (Figure 3C). Staphylococcus and streptococcus are two most common pathogenic gram positive cocci responsible for infections following cranioplasty (Figure 3C). Late complications of titanium hardware include palpability, exposure, pain and hardware malfunction, which may necessitate hardware removal. Figure 4 reports the anecdotical case of an elderly patient with a titanium cranioplasty mesh in whom tension pneumocephalus occurred five years after surgery, when a frontal scalp wound exposed the underlying titanium mesh.

### Brain drains, shunts and probes

**Figure 5:**
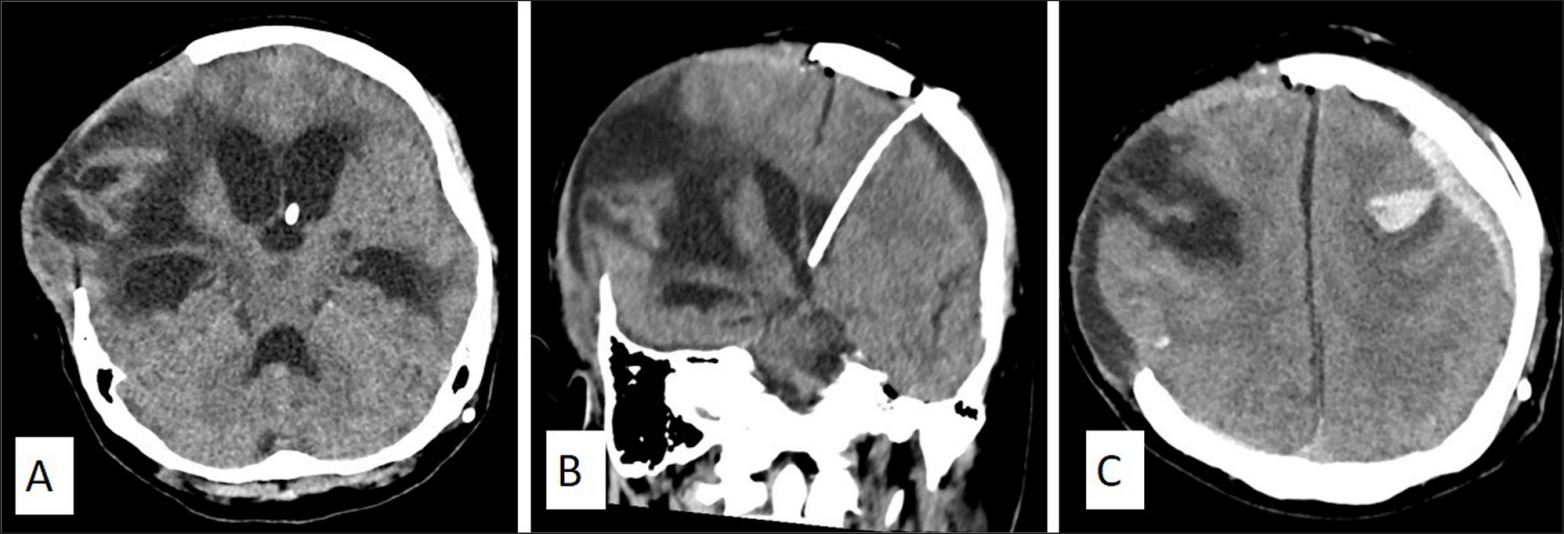
Axial brain CT of a 45-year-old woman who had right decompressive hemicraniectomy for evacuation of a right basal ganglia hemorrhage. She developed hydrocephalus for which a left frontal external ventricular catheter was inserted **(A)**. Coronal CT **(B)** shows post catheterization improvement of the hydrocephalus **(B)**. During removal of the ventricular catheter, the procedure was complicated by development of an acute left subdural and intraparenchymal hemorrhage along the catheter track seen on the axial CT **(C)**.

**Figure 6:**
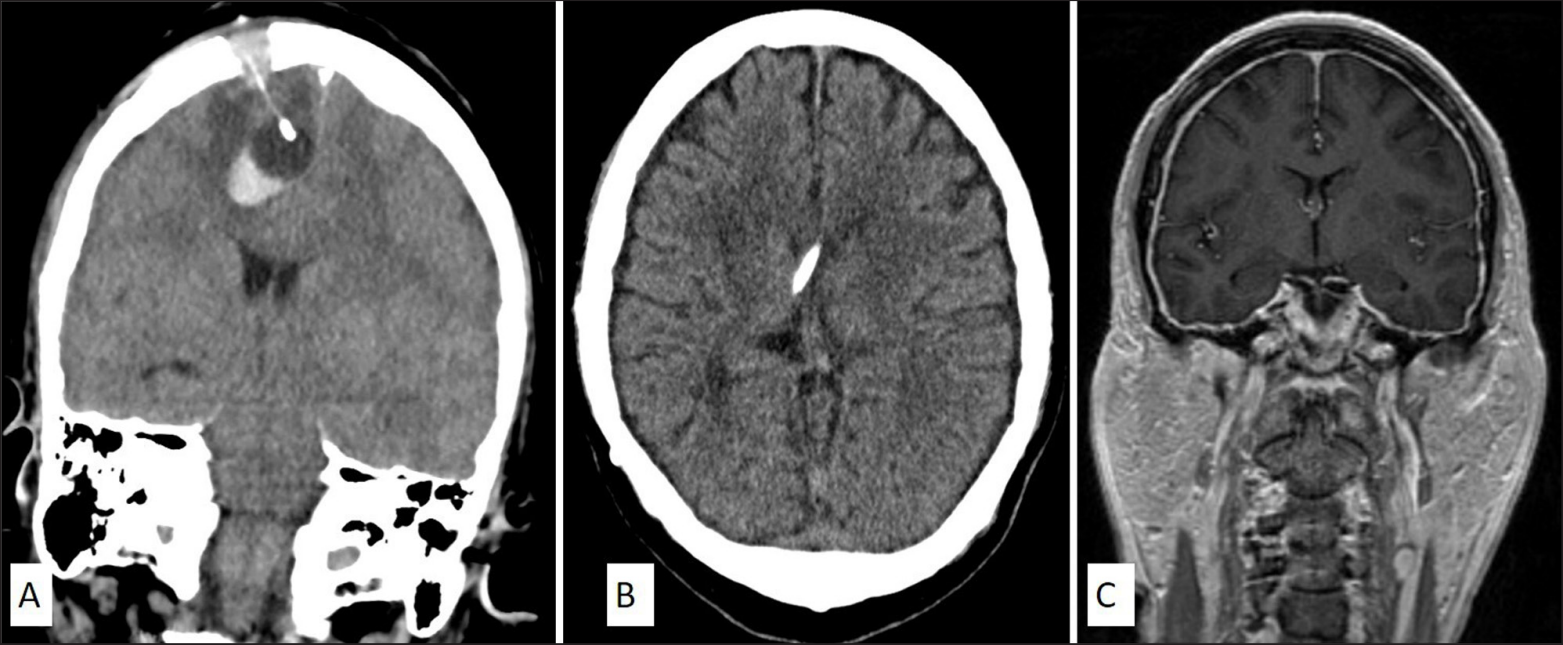
Coronal CT image of a 57-year-old man with severe brain injury who underwent insertion of a right frontal intracranial pressure (ICP) monitoring probe **(A)** demonstrates an acute intraparenchymal hematoma at the tip of the ICP monitoring probe. Axial CT brain of a 54-year-old man **(B)** shows ventricular catheter of a right parietal ventriculoperitoneal shunt abutting the septum pellucidum. The ventricles are slit-like. Coronal post-contrast T1-Weighted MRI of the man **(C)** confirms slit-like ventricles along with pachymeningeal thickening and enhancement. This is consistent with intracranial hypotension due to over-drainage of CSF.

**Figure 7:**
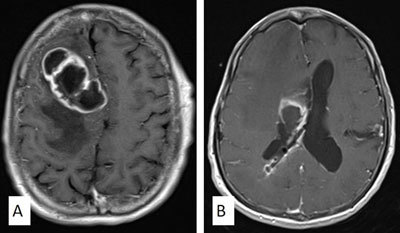
Axial post-contrast T1-weighted MRI of the brain reveals rim-enhancing collections in the right frontal lobe **(A)** and the right lateral ventricle **(B)**. In addition, there is linear enhancement along the right parietal approached ventricular catheter. These findings are compatible with shunt-related ventriculitis and cerebral abscess.

These are among the most common cranial implanted materials. Immediate postoperative complications are hemorrhages along the shunt tracks (Figures 5 and 6A) particularly in patients with history of bleeding diathesis. Early or intermediate shunt-related infections may lead to ventriculitis and abscess formation (Figure 7). Staphylococcus aureus and streptococci are the usual pathogens, particularly vulnerable to the premature, infants and immunocompromised.

Early complications are mechanical dysfunctions from shunt obstruction or disconnection leading to hydrocephalus. Dysfunctions of programmable shunt valves may necessitate adjustment of the valve threshold. Over-shunting of the cerebrospinal fluid (CSF) may result in slit-like ventricles and intracranial hypotension (Figure 6). By adjusting or increasing the valve threshold, the patient’s ventricular size may improve with subsequent improvement of symptoms. If shunt failure persists despite these measures, surgical shunt revision may be necessary.

### Deep Brain Stimulator (DBS)

**Figure 8:**
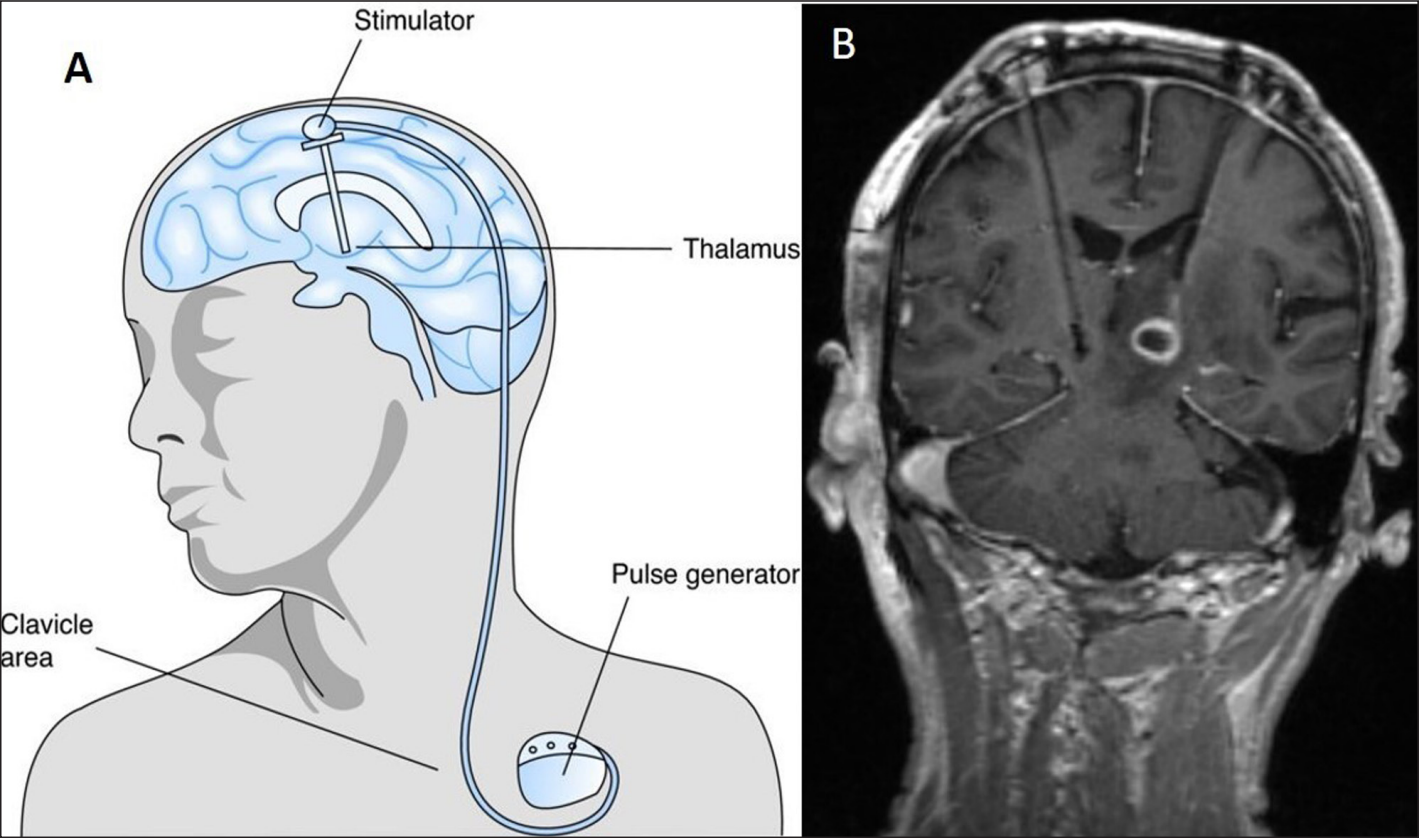
A 64-year-old man with medically intractable Parkinsonism underwent bilateral deep brain stimulator (DBS) insertion. The left stimulator electrode is connected to a battery-powered internal pulse generator in a subcutaneous pouch in the left pectoral region **(A)**. A few weeks after surgery, he developed fever and headache. Coronal post-contrast T1-weighted MRI **(B)** reveals a rim enhancing lesion at the tip of a DBS electrode in the left subthalamic region consistent with an intraparenchymal abscess. It is associated with marked perilesional oedema. Surgical removal of the infected DBS stimulator electrode was subsequently performed. The culture obtained from the removed electrode grew Staphylococcus Aureus. He was given appropriate treatment with intravenous antibiotics for six weeks.

DBS is inserted to treat medically intractable Parkinson disease. The tips of DBS electrodes are ideally positioned in the subthalamic nuclei. The electrodes are subsequently tunneled under the skin to the chest to be connected to a battery powered internal pulse generator (IPG) in a subcutaneous pouch (Figure 8). Intermediate and late post-operative complications may occur from electrode disconnection. Occasionally, infections may occur along the DBS tracks; most worrisome of all is intracranial abscess formation, which may necessitate implant removal and administration of long-term appropriate antibiotics (Figure 8).

The most common hardware-related complications are infections (5.12%), electrode breakage (0.94%), lead migration or misplacement (1.6%), and stricture formation (1.9%), fracture or failure of the lead or other parts of the implant (1.46% and 0.73%, respectively), IPG malfunctions (1.1%), and skin erosions (0.5%) [3,4].

### Endovascular treatment (EVT) materials and surgical clips

Endovascular detachable coils have been widely used for treatment of intracranial aneurysms while liquid agents (include N-butyl cyanoacrylate and Onyx) are being used for treatment of arteriovenous malformations. Artifacts from these embolization materials and clips may pose some challenges during postoperative surveillance.

## Conclusion

Postoperative imaging can be challenging and confounded by postoperative changes and implant-related complications. Awareness of these complications are paramount to clinicians and radiologists during postoperative surveillance.

## References

[1] Lied, B, Sundseth, J, Helseth, E. Immediate (0–6 h), early (6–72 h) and late (>72 h) complications after anterior cervical discectomy with fusion for cervical disc degeneration; discharge six hours after operation is feasible. Acta Neurochir (Wien). 2008; 150(2): 111–8; discussion 118. DOI: 10.1007/s00701-007-1472-y 18066487

[2] Jeyaraj, P. Importance of Early Cranioplasty in Reversing the “Syndrome of the Trephine/Motor Trephine Syndrome/Sinking Skin Flap Syndrome”. J Maxillofac Oral Surg. 2015; 14(3): 666–73. DOI: 10.1007/s12663-014-0673-1 26225060 PMC4510081

[3] Jitkritsadakul, O, Bhidayasiri, R, Kalia, SK, Hodaie, M, Lozano, AM, Fasano, A. Systematic review of hardware-related complications of Deep Brain Stimulation: Do new indications pose an increased risk? Brain Stimul. 2017; 10(5): 967–976. DOI: 10.1016/j.brs.2017.07.003 28739219

[4] Boviatsis, EJ, Stavrinou, LC, Themistocleous, M, Kouyialis, AT, Sakas, DE. Surgical and hardware complications of deep brain stimulation. A seven-year experience and review of the literature. Acta Neurochir (Wien). 2010; 152(12): 2053–62. DOI: 10.1007/s00701-010-0749-8 20658301

